# Structure-sensitive epoxidation of dicyclopentadiene over TiO_2_ catalysts†

**DOI:** 10.1039/d2cc05305e

**Published:** 2022-12-19

**Authors:** Sang-Ho Chung, G. Hwan Park, Niels Schukkink, Hyoyoung Lee, N. Raveendran Shiju

**Affiliations:** a Van’t Hoff Institute for Molecular Sciences, University of Amsterdam P.O. Box 94157 1090 GD Amsterdam The Netherlands n.r.shiju@uva.nl; b Center for Integrated Nanostructure Physics, Institute for Basic Science, Sungkyunkwan University Suwon 440-746 South Korea hyoyoung@skku.edu; c Department of Chemistry, Sungkyunkwan University Suwon 440-746 South Korea

## Abstract

Epoxidation of dicyclopentadiene (DCPD) is studied on a series of TiO_2_ catalysts using hydrogen peroxide as an oxidant. DCPD derivatives have applications in several areas including polymer, pharmaceutical and pesticide products. The control of selectivity leading to the desired product is important for many of these applications. Using experimental and computational studies, we show that the surface crystalline phases of TiO_2_ play crucial roles not only in the formation of peroxo species but also in the selective epoxidation of two different C=C double bonds in DCPD.

## Abstract 2

The crystalline phase of TiO_2_ plays a crucial role in the epoxidation of DCPD-both in the activity and in the product selectivity towards mono-epoxides in cyclopentene and norbornene moiety.

## Graphical abstract

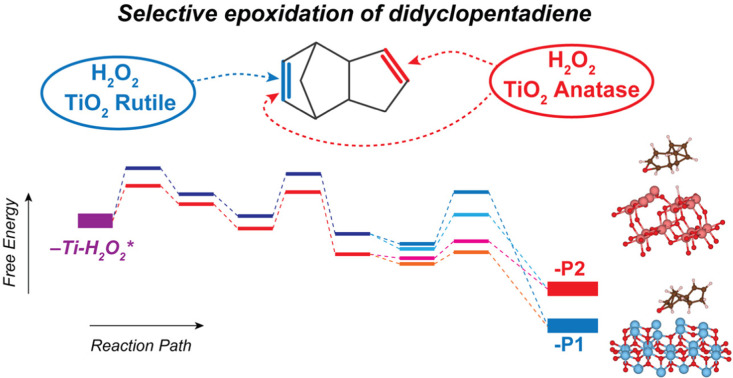

Given the versatility of the two different double bonds in its chemical structure, dicyclopentadiene (DCPD) is one of the most interesting cyclic olefin compounds. Numerous DCPD derivatives can be found in the pharmaceutical, pesticide, and polymer industries.^1^ Amongst the DCPD derivatives, DCPD epoxides find their main uses in adhesives and insulation materials.^2^

Some heterogeneous catalysts have been studied for DCPD epoxidation, such as H_3_PW_12_O_40_ on SBA-15 and on chloromethylated polystyrene resin,^3,4^ and the dispersion of phosphotungstic acid is responsible for the catalytic performance.^4^ Metal complexes intercalated in Zn/Al layered double hydroxide structures (Sulfonate-salen-M^III^, M = Mn or Fe) showed higher activity than Fe.^5^ Besides the catalytic activity (conversion rate of DCPD), product selectivity is another crucial factor in the epoxidation of DCPD. The epoxidation of DCPD yields two different mono-epoxides (*endo*-4-oxatetracyclo-[6.2.1.0.^2,6^0^3,5^]undec-9-ene (P1) and *endo*-9-oxatetracyclo-[5.3.1.0.^2,6^0^8,10^]undec-3-ene (P2)), depending on the location of the epoxide group (in the cyclopentene ring or in the norbornene ring, respectively) (Fig. 1(a)). Due to the difficulty in the product separation unit, the development of a selective epoxidation of DCPD has been encouraged,^6^ but the product selectivity typically did not rely on the chemical properties of the metal center.^3–5^

**Fig. 1:**
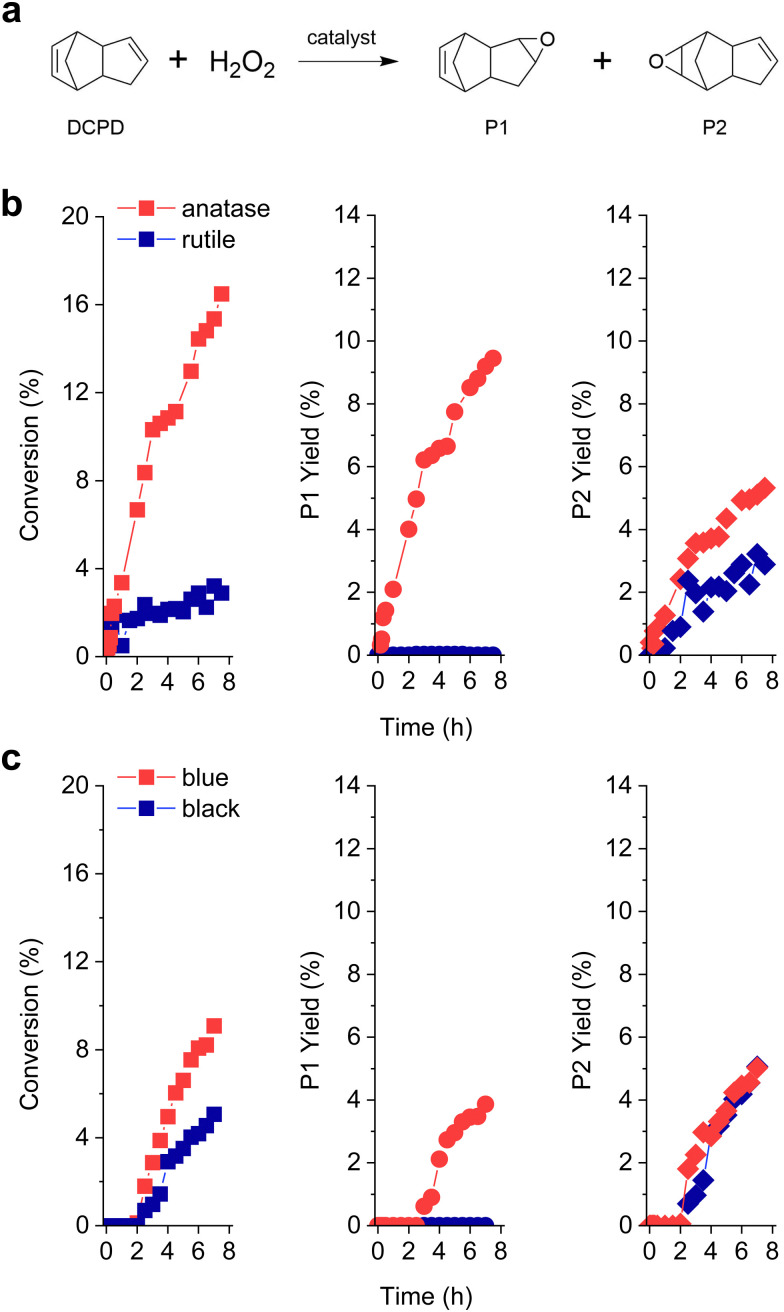
(a) Reaction scheme of DCPD epoxidation using H_2_O_2_ as an oxidant and reaction products P1 and P2. Catalytic performances of different TiO_2_ catalysts in the epoxidation of DCPD: (b) TiO_2_-anatase and TiO_2_-rutile; (c) TiO_2_-blue and TiO_2_-black.

TiO_2_ has been widely used as a catalyst for various reactions such as photocatalysis,^7^ CO oxidation,^8^ and H_2_O_2_ decomposition.^9^ In particular, the crystallinity of the TiO_2_ catalysts (anatase and rutile) is responsible for their geometric and electronic properties^10^ and plays key roles in view of the product selectivities in the reactions of the decomposition of hydrogen sulphide^11^ and the photo-oxidation of water.^12^

Our preliminary results demonstrated that the molecular oxygen (as well as the dissolved oxygen in the liquid phase) could not activate the double bonds of DCPD in methanol at 333 K. For example, insignificant conversion of DCPD (<1%) was obtained at 9 bar of pure oxygen gas in an autoclave. Thus, we studied selective DCPD epoxidation on a series of TiO_2_ catalysts using hydrogen peroxide (H_2_O_2_) as an oxidant. Indeed, the epoxidation of cyclic olefin can proceed with H_2_O_2_ in two sequential steps: (i) the formation of peroxo-species on the metal sites and (ii) the oxygen transfer from the surface to an olefin to form an epoxide.^13^

Fig. 1(b) displays the DCPD epoxidation results of two different crystalline phases of TiO_2_. TiO_2_-anatase effectively converted DCPD into the related mono-epoxides, showing *ca.* 2 times higher activity than TiO_2_-rutile (*C*_DCPD_ = 13% and 7% in 6 h, respectively). The selectivity of the epoxidation products (towards P1 and P2) is greatly influenced by the crystalline phase of the TiO_2_ catalysts. For example, on TiO_2_-anatase, both P1 and P2 were produced with higher selectivity toward P1 (Table 1). This indicates that the double bond in the cyclopentene ring is preferably reacted on the TiO_2_ anatase phase, which is in line with the previous results over the Ti-incorporated SBA-15 catalyst.^14,15^ The DCPD di-epoxide was not observed in this study, suggesting that the epoxidation sites were utilized for the reactant (DCPD) and not occupied by the mono-epoxides, similar to the results of Bhattacharjee *et al.*^5^ Interestingly, despite its lower catalytic performance, TiO_2_-rutile solely produced P2, *i.e.*, the double bond in the norbornene moiety is selectively reacted to form mono-epoxide.

**Table 1:** BET surface area and average pore size for different TiO_2_ catalysts, and the catalytic performance results for the epoxidation of DCPD

Catalyst	BET surface area (m^2^ g^−1^)	Average pore size (nm)	Normalized activity^a^ (mol_DCPD_ m^−2^ h^−1^)	*S*_P1/P2_
TiO_2_-anatase	60.8	21.8	28	1.7
TiO_2_-rutile	2.4	16.8	138	0
TiO_2_-blue	58.6	29.2	37	1.1
TiO_2_-black	2.6	16.3	350	0

a For TiO_2_-anatase and TiO_2_-rutile, the normalized activity was calculated in 6 h of reaction. For TiO_2_-blue and TiO_2_-black, the activity values were calculated in 8 h, which is *ca.* 6 h from the point of observed conversion.

We further prepared two additional TiO_2_ catalysts (TiO_2_-blue and TiO_2_-black),^16–18^ and the colors of the catalysts are attributed to the oxygen vacancies, based on the crystalline phases (anatase and rutile) (Fig. S1, ESI†).^19^ At the initial reaction stage (until *ca.* 2 h), no conversion of DCPD was observed over TiO_2_-blue and TiO_2_-black. This suggests that (i) DCPD is not reactive with H_2_O_2_ in solution and (ii) a certain delay (or lag-phase) is necessary to initiate DCPD epoxidation over the catalysts with oxygen vacancies. Since the textural properties of TiO_2_-blue and TiO_2_-black are similar to those of TiO_2_-anatase and TiO_2_-rutile, respectively (Table 1 and Fig. S1, S2, ESI,† as also reported by previous works^16–18,20^), we expected that the observed lag-phase is related to the surface oxygen vacancies, which might need to be modified by the oxygen from H_2_O_2_. In terms of the surface area normalized activity (Table 1), TiO_2_-blue and TiO_2_-black showed superior catalytic performance compared to TiO_2_-anatase and TiO_2_-rutile. We expect that the few nanometer layers of the disordered TiO_2_ surface^21^ can be attributed to the enhanced epoxidation performance. Similarly, the formation of reactive oxygen species is preferred on the amorphous ZrO_2_ than the crystalline monoclinic-ZrO_2_.^22^ Amorphous Nb_2_O_5_ and Ta_2_O_5_ also showed higher performance in the catalytic oxidation of glycerol and cyclohexene than their crystalline forms.^23,24^ For TiO_2_-blue, both P1 and P2 were produced with higher selectivity to P1 than P2. We suggest that the newly formed oxygen functionalities are highly reactive to convert the double bond in the norbornene ring as well as the one in the cyclopentene ring, similar to the fact that the oxygen vacancies are known to be responsible for the altered activity in oxidation reactions.^25^ Meanwhile, only P2 was observed on TiO_2_-black, indicating that the selectivity towards P1 or P2 is largely dependent on the surface crystalline phase.

After the epoxidation of DCPD, the color of the spent TiO_2_-anatase catalyst was changed from white to yellow, indicating the formation of the additional surface oxygen functional groups on titania.^26,27^ To identify the oxygen functionalities responsible for the catalytic activity, we characterized TiO_2_ catalysts with Raman spectroscopy (Fig. 2). For TiO_2_-anatase and TiO_2_-blue, the Raman features at 148, 395, 515 and 630 cm^−1^ are attributed to the anatase phase (the modes of E_g_, B_1g_, A_1g_ or B_1g_, and E_g_, respectively).^28^ Meanwhile, the five Raman bands are observed for TiO_2_-rutile and TiO_2_-black at 140, 235, 445, 610 and 825 cm^−1^, due to the modes of B_1g_, multi-phonon process, E_g_, A_1g_ and B_2g_, respectively. After the treatment of TiO_2_ with H_2_O_2_, the formation of oxo species on TiO_2_ (yellow coloration of TiO_2_) by H_2_O_2_ was observed with the possibility of three different forms on the Ti metal centers (oxo, peroxo, and superoxo species).^29^ The O–O stretching frequencies are typically observed between 800 and 930 cm^−1^, depending on the coordination with the environment.^30^ For example, the Raman band of H_2_O_2_ is typically positioned at 880 cm^−1^.^31^ On TiO_2_-anatase, the Raman bands of peroxo species were observed at 871 cm^−1^, indicating that the peroxo species are coordinated to the Ti sites (Fig. 3(a)).^32^ On the contrary, for TiO_2_-blue, the Raman band of peroxo species was observed at a higher wavenumber (910 cm^−1^) (Fig. 2(b)), related to the perturbation of the chemical structure of the adsorbed molecule on the catalyst surface.^33^ For the rutile phase TiO_2_ catalysts (*e.g.*, TiO_2_-rutile and TiO_2_-black), however, no additional Raman bands are observed after treatment with H_2_O_2_ (Fig. 2(b) and (d)), possibly due to (i) the low surface areas of the rutile phase TiO_2_ catalysts and (ii) the short lifetime of peroxo intermediates on the rutile phase catalysts.^34^

**Fig. 2:**
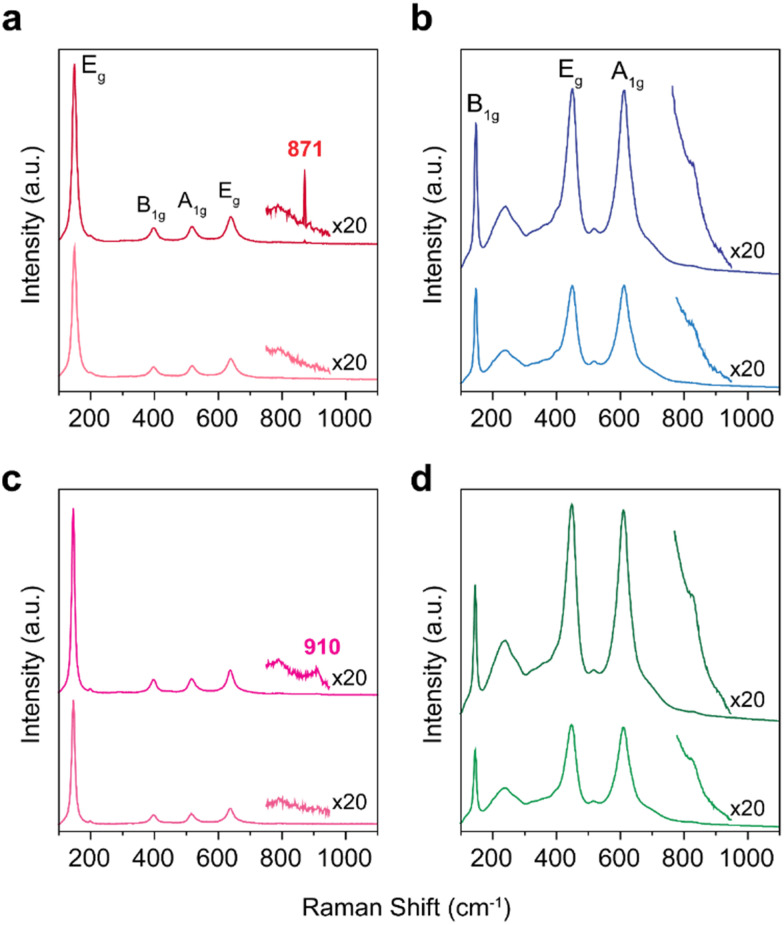
Raman spectra of TiO_2_ catalysts. (a) TiO_2_-anatase, (b) TiO_2_-rutile, (c) TiO_2_-blue and (d) TiO_2_-black. In each figure, the Raman spectra of H_2_O_2_ treated samples are displayed on top of the spectra of the untreated samples.

**Fig. 3:**
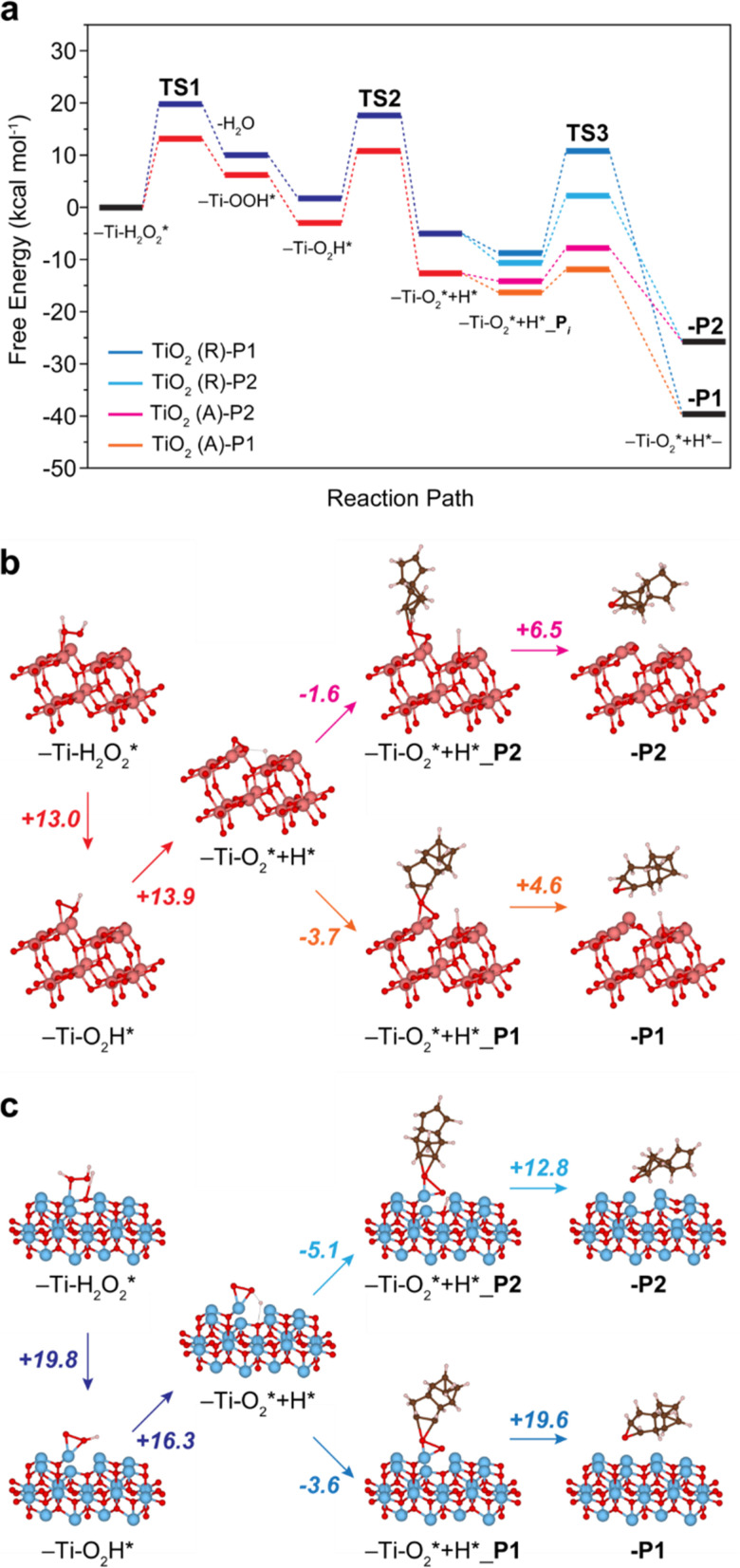
DFT-calculated free-energy profile (kcal mol^−1^) for the DCPD epoxidation with H_2_O_2_ on TiO_2_-anatase (b, orange, magenta) and TiO_2_-rutile (c, navy, blue). The free energies are calculated based on the models in Fig. S5 and S6 (ESI†). The numbers in Fig. 4b and c denote the energy barriers. Coordinates of -P2 and -P1 in (b) and (c) are provided in Tables S2–S5, ESI† as representative structures.

We have explored the reaction pathway of DCPD epoxidation and the selectivity differences among the crystalline TiO_2_ phases using density functional theory (DFT) calculation (Fig. S3 and S4, ESI†). Fig. 3(a) shows the calculated free energy profiles for DCPD epoxidation with H_2_O_2_ catalyzed by the TiO_2_-anatase and TiO_2_-rutile. Raman spectroscopy indicates that peroxo species are formed on the surface of TiO_2_, and the reaction starts from the TiO_2_ surface with adsorbed H_2_O_2_ (Ti–H_2_O_2_*). Formation of the surface peroxo species (Ti–O_2_*) proceeds *via* the generation of hydroperoxo compounds. Once the surface of TiO_2_ adsorbed H_2_O_2_, it firstly generates η^1^-coordinated Ti-hydroperoxo compounds, Ti-η^1^(OOH), following the release of a water molecule for TiO_2_-anatase and TiO_2_-rutile, which requires +13.0 (Fig. 3(b)) and +19.8 kcal mol^−1^ (Fig. 3(c)) at Transition State 1 (TS1), respectively. Subsequently, the thermodynamically more stable η^2^-coordinated Ti-hydroperoxo compound (Ti-η^2^(OOH)) is formed. The protonated Ti-peroxo species (Ti–O_2_*) can be formed from Ti-η^2^(OOH) *via* hydrogen transfer to the adjacent Ti atom overcoming moderate energy barriers of +13.9 (Fig. 3(b)) and +16.3 kcal mol^−1^ (Fig. 3(c)) at TS2 (Fig. 3(a)), respectively. Along the overall reaction steps, TiO_2_-anatase (Fig. 3(a), red color) has lower free energies than TiO_2_-rutile (Fig. 3(a), blue color), indicating a higher DCPD conversion rate of TiO_2_-anatase, which is in good agreement with our experimental results (Fig. 1(b)).

The two different C=C bonds in DCPD (cyclopentene or norbornene moiety) have different reactivity to TiO_2_ surface structures. Thus, the selectivity towards P1 or P2 strongly depends on the direction of oxygen transfer from Ti-peroxo (Ti–O_2_*). Adsorption of the DCPD molecule on the surface of TiO_2_-rutile is exothermic for both cyclopentene and norbornene with a very small energy difference (−3.6 and −5.1 kcal mol^−1^ (Fig. 3(c)), respectively). However, O–O bond cleavage with cyclopentene requires a significantly higher energy barrier compared with norbornene (+19.6 and +12.8 kcal mol^−1^ (Fig. 3(c)) at TS3 (Fig. 3(a)), respectively). This leads to the selective, one-sided oxygen transfer to the C=C double bond in norbornene, which yields nearly 100% P2 selectivity. On the other hand, in the case of TiO_2_-anatase, P1 is preferably formed since the adsorption towards the norbornene moiety requires a higher energy barrier for O–O cleavage than cyclopentene (+6.5 and +4.6 kcal mol^−1^, respectively) (see Fig. 3(b)).

In summary, the surface crystalline phase of TiO_2_ catalysts plays a crucial role in selective DCPD epoxidation, not only in the conversion rate of DCPD but also in the product selectivity towards mono-epoxides in cyclopentene and the norbornene moiety. Moreover, the surface oxygen vacancies of TiO_2_-blue and TiO_2_-black are responsible for the lag-phases at the initial reaction stages, indicating that the formation of the surface peroxo functionalities (Ti–O_2_*) is the rate-determining step.

We acknowledge the Dutch research council (NWO) for the funding (LIFT project 731.017.412), the Institute for Basic Science (IBS-R011-D1), and the Korea Medical Device Development Fund (KMDF_PR_20200901_0004). We thank V. Lachman, N. J. Geels, P. F. Collignon and M.C. Mittelmeijer–Hazeleger (UvA) for the technical support.

## Conflicts of interest

There are no conflicts to declare.

## Supplementary Material

CC-059-D2CC05305E-s001
