# Supplementary material for: Structure-sensitive epoxidation of dicyclopentadiene over TiO2 catalysts
Source: Chem Commun (Camb). 2022 Dec 19;59(6):756–9. doi: 10.1039/d2cc05305e (PMC9844381; doi:10.1039/d2cc05305e)
Supplement: CC-059-D2CC05305E-s001 [file CC-059-D2CC05305E-s001.pdf]

Electronic Supplementary Material (ESI) for Chemical Communications.
This journal is © The Royal Society of Chemistry 2022

Supplementary Information

Structure-sensitive epoxidation of dicyclopentadiene over TiO_2 catalysts†

Sang-Ho Chung,[a‡] G. Hwan Park,[b,c‡] Niels Schukkink,[a] Hyoyoung Lee,[b,c*], N. Raveendran Shiju[a*]

[a] Van 't Hoff Institute for Molecular Sciences, University of Amsterdam, P.O. Box 94157, 1090 GD Amsterdam, The Netherlands.

[b] Center for Integrated Nanostructure Physics, Institute for Basic Science, Sungkyunkwan University, Suwon 440-746, South Korea.

[c] Department of Chemistry, Sungkyunkwan University, Suwon 440-746, South Korea.

‡The authors contributed the manuscript equally.

[*] E-mail: (N.R.S.) n.r.shiju@uva.nl; (H.L.) hyoyoung@skku.edu

Methods

Preparation of TiO_2 catalysts. The detailed preparation methods for TiO_2–anatase, rutile, blue and black samples are described in the previous publication.[1] Briefly, the pristine P25 (Degussa) was dispersed in 50 mL of 1 M Li–ethylenediamine solution (Sigma–Aldrich). The solution was stirred for 7 days under inert atmosphere. After the reaction, the solution was cooled down and HCl (35%, OCI Ltd.) was sequentially added to achieve pH 7. Finally, the resulting TiO_2–blue was filtered with deionized water and ethanol to remove the remaining Li^+, Cl^-, and dried overnight under vacuum. TiO_2–black was synthesized in the same way as TiO_2-blue was, and only TiO_2–rutile (Sigma–Aldrich) was used instead of pristine P25.

Characterisation. Powder X-ray diffraction was carried out using Rigaku Miniflex–II with a scanning range of 2θ = 5–90° at 2.5°/min (Cu Kα, λ = 1.5406Å). The composition of anatase phase in TiO_2–rutile sample was calculated using the equation (anatase% = $[1+1.265(I_R/I_A)]^{-1}$)[2]. The specific surface area of the catalysts was determined using Brunauer-Emmett-Teller (BET) on a Micromeritics ASAP 2020. Raman spectra were measured using Renishaw inVia dispersive spectrometer equipped with a CCD detector. The Raman scattered light was collected with a 50x Olympus objective in the spectral range of 100–2000 cm^{-1}. Two laser lines (785 and 514 nm, respectively) were checked to optimise the quality of the signals. The spectra were recorded at a resolution of 2 cm^{-1} at ambient conditions, and the scans were repeatedly collected for each sample to ensure a good signal–to–noise ratio. Before Raman spectroscopy measurements, two sets of samples were prepared (before and after treatment with H_2O_2). For the H_2O_2 treated samples, H_2O_2 was added to the catalyst with solvent. The samples were dried at 333 K.

Catalytic tests. For DCPD epoxidation, 100 mg of TiO_2 catalysts and were firstly added to a round-bottom flask containing 50 mL of 15 mM of DCPD–methanol solution. DCPD (95%) and methanol (99.8%) were purchased from Alfa Aesar and Biosolve B.V., respectively. When temperature of the mixture was reached to 333 K, 4.3 mL of H_2O_2 (30% w/v, Fischer Scientific) was added and it is considered as the starting point of the reaction (t = 0). Methyl benzoate (99%, Sigma-Aldrich) was used as internal standard. The reaction was monitored by periodic sampling. The trace water in the liquid samples was removed adding excess of anhydrous magnesium sulphate before GC analysis. The reaction mixtures were analysed by GC-FID (Perkin-Elmer Clarius 580) with HP–5 column (30 m × 0.25 mm × 0.2 mm). Without TiO_2 catalysts, less than 1% of DCPD conversion was observed. If necessary, the liquid product was analysed by GC–MS with HP–5 column. The electron ionisation method was applied with filament ionizing voltage of 70 V and the related mass spectra were collected on an AccuTOF LC, JMS–T100LP Mass spectrometer (JEOL).

Computational details. We have employed the first-principles[3,4] to perform spin–polarization density functional theory (DFT) calculations within the generalized gradient approximation (GGA) using the Perdew–Burke–Ernzerhof (PBE) formulation.[5] The PBE is a commonly used computational functional which can qualitatively judge the reaction rates and theoretically explain some preferred reaction pathways.[6,7] Despite the relatively high accuracy of PBEsol or revM06-L, the whole trends on epoxidation reactions on TiO_2 still could be explained by using PBE. We have chosen the projected augmented wave (PAW) potentials[8,9] to describe the ionic cores and take valence electrons into account using a plane wave basis set with a kinetic energy cutoff of 450 eV. Partial occupancies of the Kohn−Sham orbitals were allowed using the Gaussian smearing method and a width of 0.05 eV. The

electronic energy was considered self–consistent when the energy change was smaller than 10^{-6} eV. A geometry optimization was considered convergent when the energy change was smaller than 0.05 eV $Å^{-1}$. In addition, for the Ti atoms, the U schemes need to be applied, and the U has been set as 3.86 eV. The vacuum spacing in a direction perpendicular to the plane of the structure is 15 Å for TiO_2 surface. The Brillouin zone integration is performed using 3×3×1 Monkhorst–Pack k–point sampling for a structure. Grimme's DFT–D3 methodology[10] was used to describe the dispersion interactions among all the atoms in adsorption models. Finally, the adsorption energies (E_{ads}) were calculated as $E_{ads} = E_{ad/sub} - E_{ad} - E_{sub}$, where $E_{ad/sub}$, E_{ad}, and E_{sub} are the total energies of the optimized adsorbate/substrate system, the adsorbate in the gas phase, and the clean substrate, respectively. The free energy was calculated using the equation:

$$G = E + ZPE - TS \quad (1)$$

where G, E, ZPE and TS are the free energy, total energy from DFT calculations, zero-point energy and entropic contributions (T was set to be 300 K), respectively. In this study, exo-DCPD, which is more thermodynamically stable than endo-DCPD, was employed as a model structure in DFT calculations and the following diastereomeric products after epoxidation on double bonds of cyclopentene and norbornene is described in Figure S7.

Supplementary note 1. The composition of the TiO_2 phases (the ratio between rutile and anatase) was found to be 97% rutile by XRD analysis (Fig. 2a). *i.e.*, there is still 3% of anatase phase in the TiO_2–rutile catalyst. Zhang et al. recently reported that, the most of the surface of anatase TiO_2 nanofibers is covered with rutile phase after the thermal treatment (anatase–rutile core–shell structure).[11] We suggest that the dominant rutile phase is fully–covering the surface of the catalyst and the inner anatase phase

could not participate in the epoxidation of DCPD and, correspondingly, in the product distribution. From the equation of intensity ratio of Raman spectra $(1/[1+0.038(I_{140}/I_{440})])$[12], the anatase composition was calculated from the Raman spectra of TiO_2–rutile and TiO_2–black, and it was found 4 % of anatase phase is present in the samples, in agreement with the results of the anatase composition determined by XRD analysis.

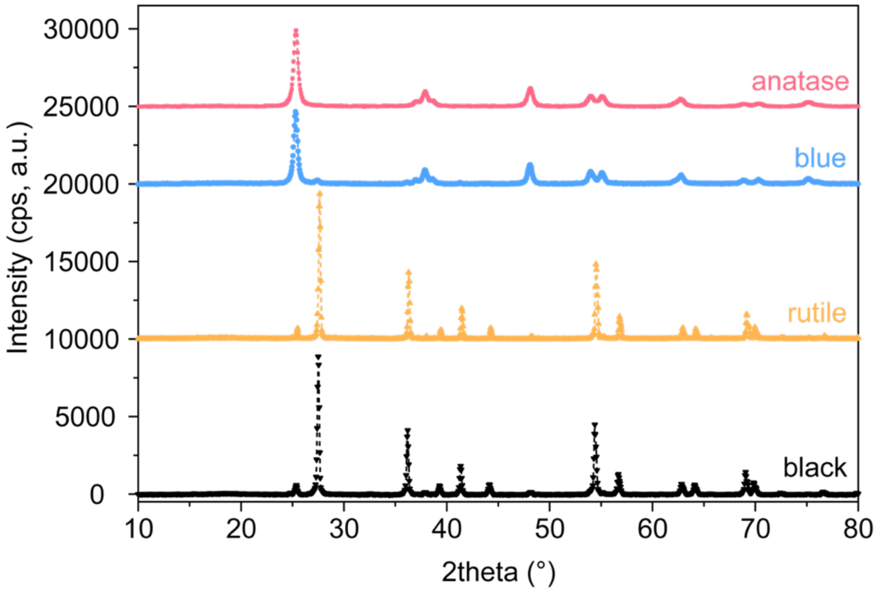

Figure S1. Powder XRD of TiO_2 catalysts.

Table S1. Lattice parameters, unit cell volume, average crystallite size of TiO_2 catalysts from XRD analysis.

Sample	Phase	Space group	Average crystallite size[1] (nm)	Lattice parameters (Å)			Unit cell volume (Å^3)
				a	b	c	
TiO_2-anatase	Anatase	I41/amd	14.1	3.784	3.784	9.497	136.0
TiO_2-rutile	Rutile	P42/mnm	15.2	4.574	4.574	2.953	61.8
TiO_2-blue	Anatase	I41/amd	40.2	3.784	3.784	9.509	136.1
TiO_2-black	Rutile	P42/mnm	38.4	4.585	4.585	2.959	62.2

[1]The average crystallite size (D_{hkl})was calculated using Scherrer equation ($D_{hkl} = K\lambda/(B_{hkl}\cos\theta)$) with the crystallite-shape factor of 0.94.

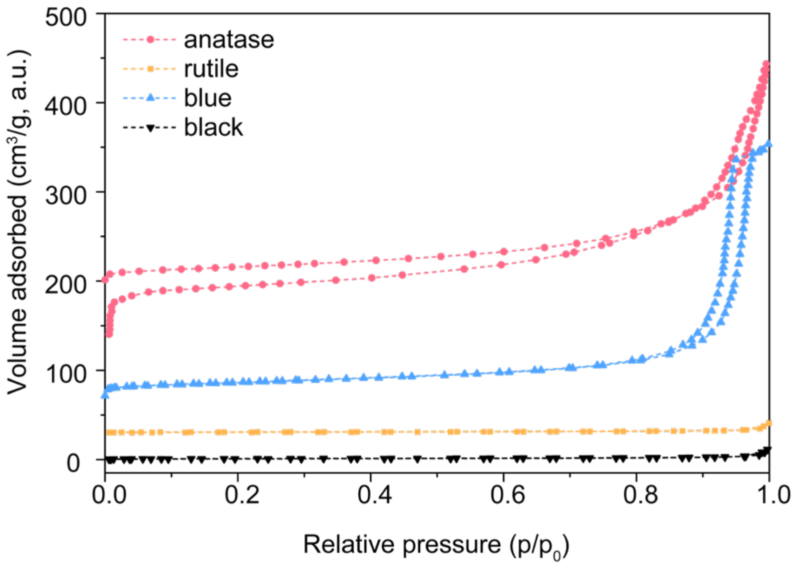

Figure S2. N_2 isotherms of TiO_2 catalysts.

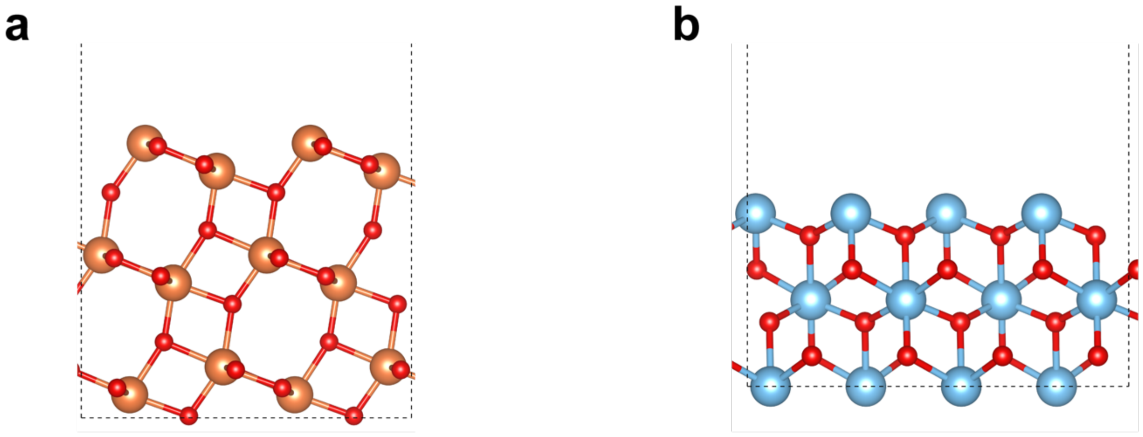

Figure S3. The structure models of **a,** TiO_2–anatase (101) slab and **b,** TiO_2–rutile (101) slab.

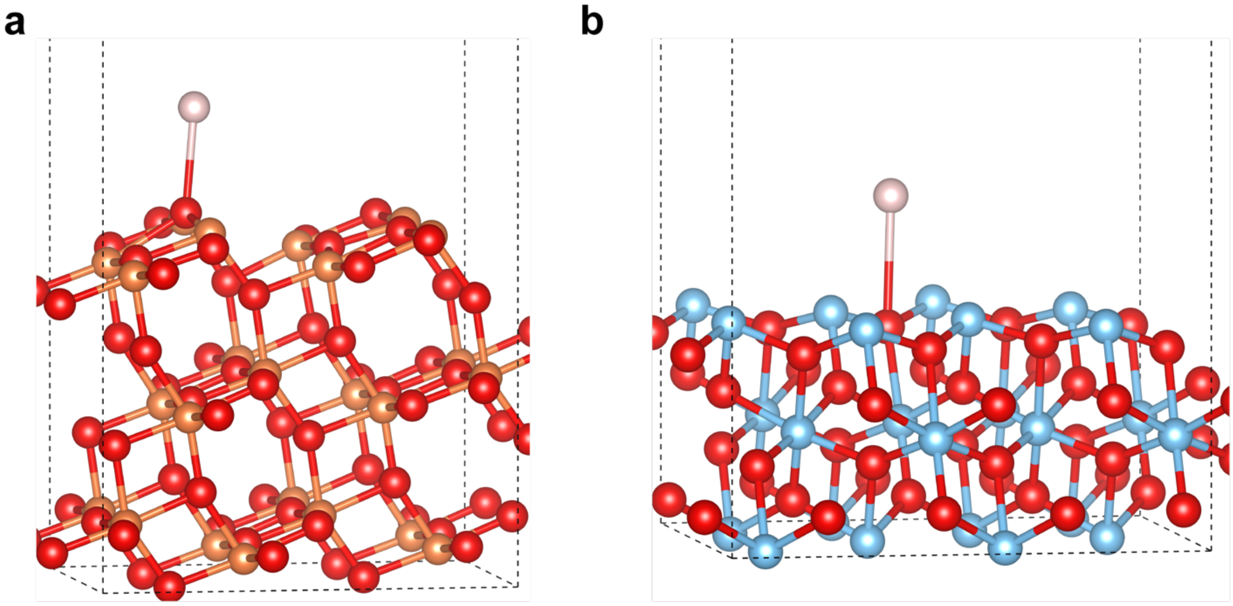

Figure S4. The structure models with surface –OH of **a,** TiO_2–anatase (101) slab and **b,** TiO_2–rutile (101) slab.

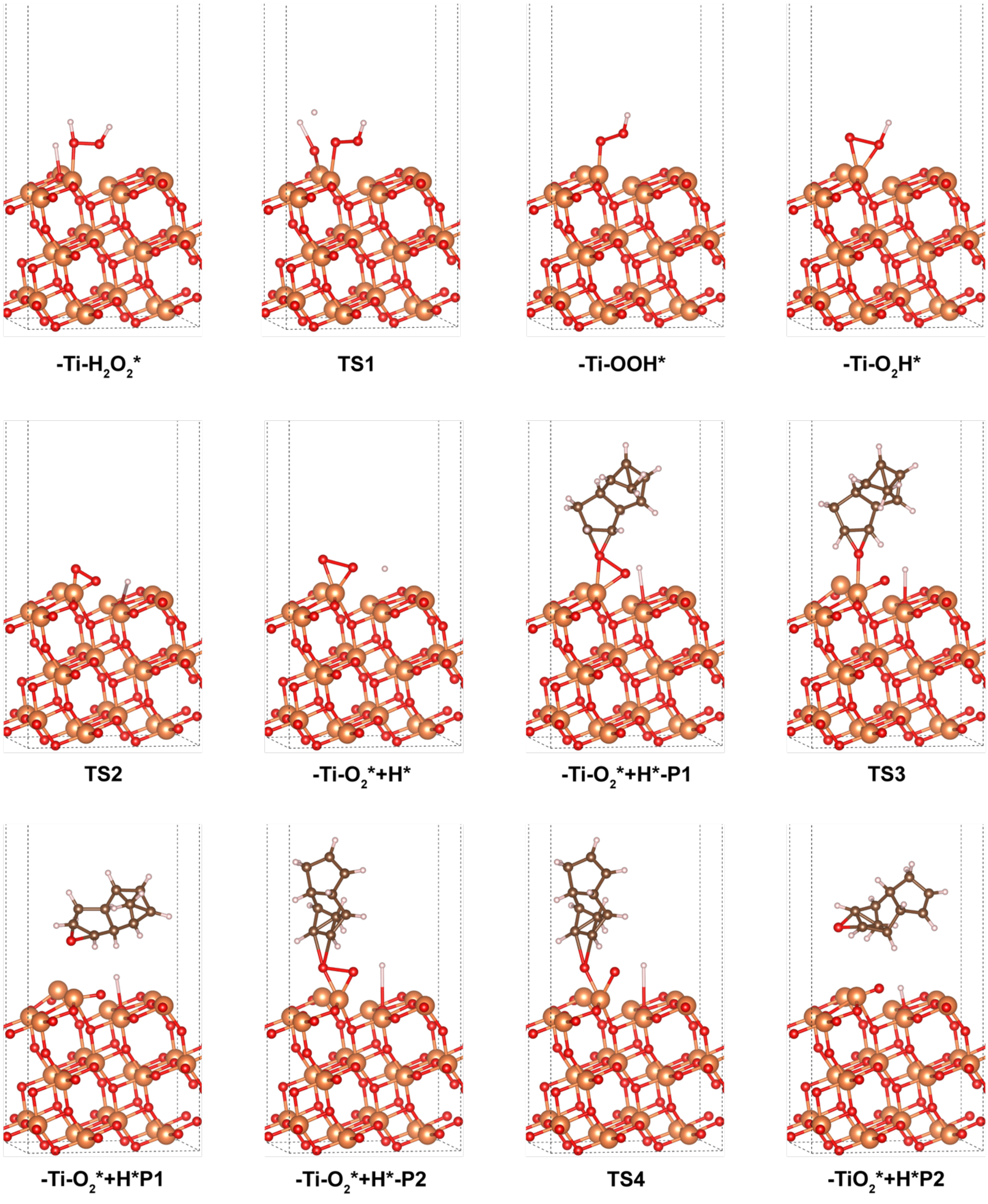

Figure S5. The configurations of the intermediates and transition states involved in the DCPD epoxidation on TiO_2–anatase (101) slab.

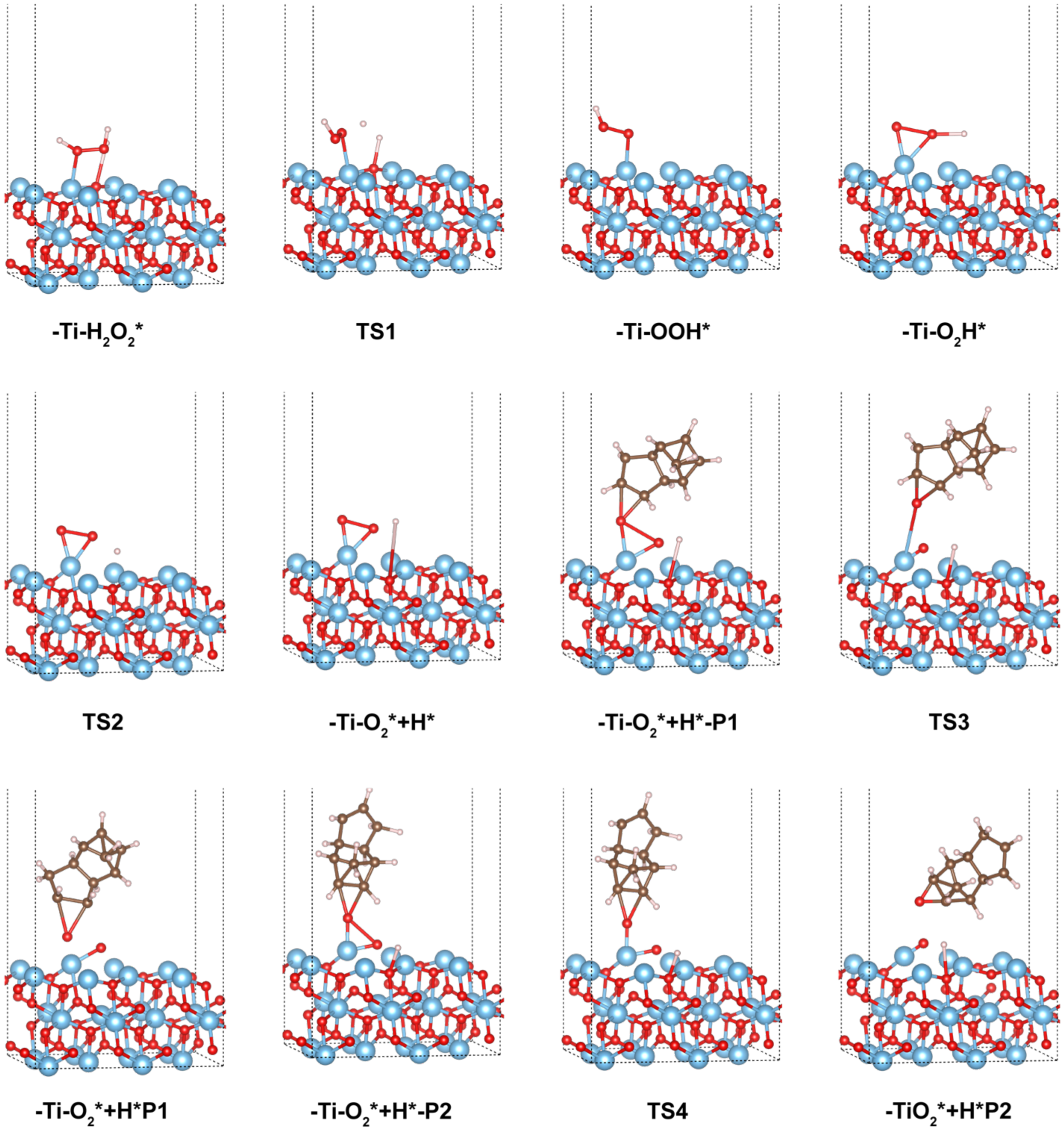

Figure S6. The configurations of the intermediates and transition states involved in the DCPD epoxidation on TiO_2–rutile (101) slab.

Table S2. Coordinates of the -P1 state from TiO_2-Anatase.

**Ti**	0.42961	0.13889	0.07447	**O**	0.2507	0.63938	0.11084
**Ti**	0.56169	0.13889	0.23771	**O**	0.38278	0.63938	0.27409
**Ti**	0.71346	0.1323	0.42496	**O**	0.32554	0.63938	0.00272
**Ti**	0.64659	0.38889	0.03366	**O**	0.45763	0.63938	0.16597
**Ti**	0.77867	0.38889	0.1969	**O**	0.58971	0.63938	0.32921
**Ti**	0.91076	0.38889	0.36015	**O**	0.60856	0.63938	0.04353
**Ti**	0.92961	0.38889	0.07447	**O**	0.74065	0.63938	0.20678
**Ti**	0.06169	0.38889	0.23771	**O**	0.87273	0.63938	0.37002
**Ti**	0.19378	0.38889	0.40096	**O**	0.46768	0.88938	0.07003
**Ti**	0.14659	0.13889	0.03366	**O**	0.59976	0.88938	0.23328
**Ti**	0.27867	0.13889	0.1969	**O**	0.72115	0.89383	0.39021
**Ti**	0.41918	0.13395	0.36856	**O**	0.7507	0.88938	0.11084
**O**	0.2507	0.13938	0.11084	**O**	0.88278	0.88938	0.27409
**O**	0.38278	0.13938	0.27409	**O**	0.82554	0.88938	0.00272
**O**	0.32554	0.13938	0.00272	**O**	0.95763	0.88938	0.16597
**O**	0.45763	0.13938	0.16597	**O**	0.08971	0.88938	0.32921
**O**	0.58971	0.13938	0.32921	**O**	0.10856	0.88938	0.04353
**O**	0.60856	0.13938	0.04353	**O**	0.24065	0.88938	0.20678
**O**	0.74065	0.13938	0.20678	**O**	0.36848	0.89142	0.36687
**O**	0.87273	0.13938	0.37002	**O**	0.96768	0.63938	0.07003
**O**	0.46768	0.38938	0.07003	**O**	0.09976	0.63938	0.23328
**O**	0.59976	0.38938	0.23328	**O**	0.23396	0.63815	0.39863
**O**	0.7507	0.38938	0.11084	**O**	0.73015	0.85879	0.58419
**O**	0.88278	0.38938	0.27409	**O**	0.54385	0.75629	0.43059
**O**	0.82554	0.38938	0.00272	**H**	0.40945	0.42504	0.46792
**O**	0.95763	0.38938	0.16597	**C**	-0.7504	-0.26633	0.65842
**O**	0.08971	0.38938	0.32921	**C**	-0.70018	-0.3591	0.71425
**O**	0.10856	0.38938	0.04353	**C**	-0.5564	-0.3243	0.71804
**O**	0.24065	0.38938	0.20678	**C**	-0.50467	-0.42936	0.66232
**O**	0.37691	0.38648	0.37528	**C**	-0.54089	-0.30611	0.6062
**O**	0.96768	0.13938	0.07003	**C**	-0.63825	-0.16911	0.62932
**O**	0.09976	0.13938	0.23328	**C**	-0.41401	-0.21434	0.59618
**O**	0.23157	0.13637	0.40388	**C**	-0.30512	-0.31558	0.62487
**Ti**	0.42961	0.63889	0.07447	**C**	-0.35482	-0.47136	0.66216
**Ti**	0.56169	0.63889	0.23771	**C**	-0.62135	-0.20455	0.68193
**Ti**	0.69715	0.72314	0.41992	**H**	-0.8551	-0.25399	0.64454
**Ti**	0.64659	0.88889	0.03366	**H**	-0.76015	-0.43041	0.75029
**Ti**	0.77867	0.88889	0.1969	**H**	-0.51209	-0.34946	0.76521
**Ti**	0.91076	0.88889	0.36015	**H**	-0.55894	-0.55925	0.66391
**Ti**	0.92961	0.88889	0.07447	**H**	-0.56314	-0.37899	0.56158

**Ti**	0.06169	0.88889	0.23771	**H**	-0.65927	-0.05015	0.59901
**Ti**	0.19378	0.88889	0.40096	**H**	-0.40138	-0.08741	0.56893
**Ti**	0.14659	0.63889	0.03366	**H**	-0.20076	-0.27279	0.62316
**Ti**	0.27867	0.63889	0.1969	**H**	-0.30332	-0.49934	0.70741
**Ti**	0.41076	0.63889	0.36015	**H**	-0.35349	-0.59112	0.63057
				**H**	-0.67692	-0.11279	0.71368
				**H**	-0.52185	-0.14947	0.67273

Table S3. Coordinates of the -P2 state from TiO_2-Anatase.

**Ti**	0.42961	0.13889	0.07447	**O**	0.2507	0.63938	0.11084
**Ti**	0.56169	0.13889	0.23771	**O**	0.38278	0.63938	0.27409
**Ti**	0.69578	0.13759	0.40611	**O**	0.32554	0.63938	0.00272
**Ti**	0.64659	0.38889	0.03366	**O**	0.45763	0.63938	0.16597
**Ti**	0.77867	0.38889	0.1969	**O**	0.58971	0.63938	0.32921
**Ti**	0.91076	0.38889	0.36015	**O**	0.60856	0.63938	0.04353
**Ti**	0.92961	0.38889	0.07447	**O**	0.74065	0.63938	0.20678
**Ti**	0.06169	0.38889	0.23771	**O**	0.87273	0.63938	0.37002
**Ti**	0.19378	0.38889	0.40096	**O**	0.46768	0.88938	0.07003
**Ti**	0.14659	0.13889	0.03366	**O**	0.59976	0.88938	0.23328
**Ti**	0.27867	0.13889	0.1969	**O**	0.72115	0.89383	0.39021
**Ti**	0.41918	0.13395	0.36856	**O**	0.7507	0.88938	0.11084
**O**	0.2507	0.13938	0.11084	**O**	0.88278	0.88938	0.27409
**O**	0.38278	0.13938	0.27409	**O**	0.82554	0.88938	0.00272
**O**	0.32554	0.13938	0.00272	**O**	0.95763	0.88938	0.16597
**O**	0.45763	0.13938	0.16597	**O**	0.08971	0.88938	0.32921
**O**	0.58971	0.13938	0.32921	**O**	0.10856	0.88938	0.04353
**O**	0.60856	0.13938	0.04353	**O**	0.24065	0.88938	0.20678
**O**	0.74065	0.13938	0.20678	**O**	0.36848	0.89142	0.36687
**O**	0.87273	0.13938	0.37002	**O**	0.96768	0.63938	0.07003
**O**	0.46768	0.38938	0.07003	**O**	0.09976	0.63938	0.23328
**O**	0.59976	0.38938	0.23328	**O**	0.23396	0.63815	0.39863
**O**	0.7507	0.38938	0.11084	**O**	0.79834	0.64134	0.61559
**O**	0.88278	0.38938	0.27409	**O**	0.5769	0.76657	0.44805
**O**	0.82554	0.38938	0.00272	**H**	0.40615	0.41773	0.43759
**O**	0.95763	0.38938	0.16597	**C**	-0.32987	-0.25821	0.61929
**O**	0.08971	0.38938	0.32921	**C**	-0.30764	-0.41771	0.65829
**O**	0.10856	0.38938	0.04353	**C**	-0.43696	-0.50615	0.66928
**O**	0.24065	0.38938	0.20678	**C**	-0.50522	-0.37263	0.71182
**O**	0.37691	0.38648	0.37528	**C**	-0.54638	-0.2237	0.6649
**O**	0.96768	0.13938	0.07003	**C**	-0.47215	-0.25271	0.60442
**O**	0.09976	0.13938	0.23328	**C**	-0.6846	-0.27497	0.65427
**O**	0.23157	0.13637	0.40388	**C**	-0.7339	-0.39237	0.70507
**Ti**	0.42961	0.63889	0.07447	**C**	-0.62656	-0.44352	0.74919
**Ti**	0.56169	0.63889	0.23771	**C**	-0.43593	-0.3944	0.61918
**Ti**	0.70229	0.7216	0.42505	**H**	-0.25294	-0.16974	0.59872
**Ti**	0.64659	0.88889	0.03366	**H**	-0.21056	-0.47169	0.67279
**Ti**	0.77867	0.88889	0.1969	**H**	-0.43124	-0.64905	0.68489
**Ti**	0.91076	0.88889	0.36015	**H**	-0.42886	-0.33142	0.74639
**Ti**	0.92961	0.88889	0.07447	**H**	-0.54813	-0.08558	0.68527

**Ti**	0.06169	0.88889	0.23771	**H**	-0.50437	-0.17857	0.56149
**Ti**	0.19378	0.88889	0.40096	**H**	-0.74526	-0.2272	0.61398
**Ti**	0.14659	0.63889	0.03366	**H**	-0.83518	-0.44725	0.70721
**Ti**	0.27867	0.63889	0.1969	**H**	-0.62807	-0.58469	0.76684
**Ti**	0.41076	0.63889	0.36015	**H**	-0.62801	-0.34532	0.78887
				**H**	-0.38121	-0.46137	0.58028
				**H**	-0.5418	-0.42882	0.61643

Table S4. Coordinates of the -P1 state from TiO_2-Rutile.

**Ti**	0.06077	0.13889	0	**O**	0.05791	0.73684	0.10327
**Ti**	0.41609	0.13889	0.13901	**O**	0.41324	0.73684	0.24228
**Ti**	0.2599	0.14225	0.2779	**Ti**	0.56077	0.63889	0
**Ti**	0.31077	0.38889	0	**Ti**	0.91609	0.63889	0.13901
**Ti**	0.16609	0.38889	0.13901	**Ti**	0.77142	0.63889	0.27802
**Ti**	0.02142	0.38889	0.27802	**Ti**	0.81077	0.88889	0
**O**	0.30791	0.29142	0.10327	**Ti**	0.66609	0.88889	0.13901
**O**	0.16324	0.29142	0.24228	**Ti**	0.52142	0.88889	0.27802
**O**	0.16904	0.48684	0.04894	**O**	0.80791	0.79142	0.10327
**O**	0.02436	0.48684	0.18795	**O**	0.66324	0.79142	0.24228
**O**	0.41904	0.04142	0.04894	**O**	0.66904	0.98684	0.04894
**O**	0.27436	0.04142	0.18795	**O**	0.52436	0.98684	0.18795
**O**	0.05791	0.23684	0.10327	**O**	0.91904	0.54142	0.04894
**O**	0.41324	0.23684	0.24228	**O**	0.77436	0.54142	0.18795
**Ti**	0.56077	0.13889	0	**O**	0.55791	0.73684	0.10327
**Ti**	0.91609	0.13889	0.13901	**O**	0.91324	0.73684	0.24228
**Ti**	0.77142	0.13889	0.27802	**C**	0.70895	0.43415	0.51814
**Ti**	0.81077	0.38889	0	**C**	0.68256	0.5161	0.58785
**Ti**	0.66609	0.38889	0.13901	**C**	0.54502	0.5208	0.59792
**Ti**	0.52142	0.38889	0.27802	**C**	0.5049	0.62035	0.53366
**O**	0.80791	0.29142	0.10327	**C**	0.514	0.51724	0.46398
**O**	0.66324	0.29142	0.24228	**C**	0.58777	0.38391	0.48703
**O**	0.66904	0.48684	0.04894	**C**	0.38236	0.47344	0.45641
**O**	0.52436	0.48684	0.18795	**C**	0.29941	0.57734	0.49587
**O**	0.91904	0.04142	0.04894	**C**	0.37206	0.68813	0.53971
**O**	0.77436	0.04142	0.18795	**C**	0.58375	0.41261	0.55117
**O**	0.55791	0.23684	0.10327	**H**	0.80647	0.42041	0.49857
**O**	0.91324	0.23684	0.24228	**H**	0.74416	0.57014	0.6303
**Ti**	0.06077	0.63889	0	**H**	0.50951	0.54088	0.65003
**Ti**	0.41609	0.63889	0.13901	**H**	0.57372	0.71085	0.52733
**Ti**	0.26256	0.64179	0.28884	**H**	0.55122	0.57278	0.41237
**Ti**	0.31077	0.88889	0	**H**	0.57128	0.27155	0.46392
**Ti**	0.16609	0.88889	0.13901	**H**	0.37522	0.37041	0.49037
**Ti**	0.02142	0.88889	0.27802	**H**	0.26239	0.50698	0.54246
**O**	0.30791	0.79142	0.10327	**H**	0.34073	0.69248	0.6
**O**	0.16324	0.79142	0.24228	**H**	0.36301	0.79784	0.51209
**O**	0.16904	0.98684	0.04894	**H**	0.68013	0.39084	0.57091
**O**	0.02436	0.98684	0.18795	**H**	0.52823	0.32784	0.58167
**O**	0.41904	0.54142	0.04894	**O**	0.25083	0.49859	0.4268
**O**	0.27436	0.54142	0.18795				

Table S5. Coordinates of the -P2 state from TiO_2-Rutile.

**Ti**	0.09027	0.13889	0.0014
**Ti**	0.4456	0.13889	0.14041
**Ti**	0.30092	0.13889	0.27942
**Ti**	0.34027	0.38889	0.0014
**Ti**	0.1956	0.38889	0.14041
**Ti**	0.05092	0.38889	0.27942
**O**	0.33741	0.29142	0.10467
**O**	0.19274	0.29142	0.24368
**O**	0.19854	0.48684	0.05034
**O**	0.05386	0.48684	0.18935
**O**	0.44854	0.04142	0.05034
**O**	0.30386	0.04142	0.18935
**O**	0.08741	0.23684	0.10467
**O**	0.44666	0.23891	0.25734
**Ti**	0.59027	0.13889	0.0014
**Ti**	0.9456	0.13889	0.14041
**Ti**	0.80529	0.13892	0.29121
**Ti**	0.84027	0.38889	0.0014
**Ti**	0.6956	0.38889	0.14041
**Ti**	0.5529	0.38845	0.29505
**O**	0.83741	0.29142	0.10467
**O**	0.69388	0.29557	0.26695
**O**	0.69854	0.48684	0.05034
**O**	0.55386	0.48684	0.18935
**O**	0.94854	0.04142	0.05034
**O**	0.80386	0.04142	0.18935
**O**	0.58741	0.23684	0.10467
**O**	0.9429	0.23796	0.24711
**Ti**	0.09027	0.63889	0.0014
**Ti**	0.4456	0.63889	0.14041
**Ti**	0.2871	0.6495	0.30519
**Ti**	0.34027	0.88889	0.0014
**Ti**	0.1956	0.88889	0.14041
**Ti**	0.05092	0.88889	0.27942
**O**	0.33741	0.79142	0.10467
**O**	0.19511	0.79458	0.25396
**O**	0.19854	0.98684	0.05034
**O**	0.05386	0.98684	0.18935
**O**	0.44854	0.54142	0.05034
**O**	0.30386	0.54142	0.18935
**O**	0.08741	0.73684	0.10467
**Ti**	0.59027	0.63889	0.0014
**Ti**	0.9456	0.63889	0.14041
**Ti**	0.80529	0.63892	0.29121
**Ti**	0.84027	0.88889	0.0014
**Ti**	0.6956	0.88889	0.14041
**Ti**	0.55149	0.88878	0.29791
**O**	0.83741	0.79142	0.10467
**O**	0.69571	0.795	0.26578
**O**	0.69854	0.98684	0.05034
**O**	0.55386	0.98684	0.18935
**O**	0.94854	0.54142	0.05034
**O**	0.80386	0.54142	0.18935
**O**	0.58741	0.73684	0.10467
**O**	0.94128	0.74002	0.25285
**O**	0.31838	0.46816	0.41775
**C**	0.40841	0.58471	0.46279
**C**	0.44693	0.43137	0.45405
**C**	0.55542	0.40585	0.50523
**C**	0.65589	0.49585	0.46749
**C**	0.62086	0.65433	0.49108
**C**	0.49038	0.65162	0.52145
**C**	0.70681	0.67531	0.55582
**C**	0.81054	0.56975	0.55319
**C**	0.79243	0.4604	0.49102
**C**	0.48711	0.52435	0.52449
**H**	0.32915	0.63928	0.46586
**H**	0.46559	0.3546	0.41911
**H**	0.57501	0.28815	0.51799
**H**	0.64526	0.47209	0.40573
**H**	0.64515	0.74011	0.44842
**H**	0.45595	0.74947	0.55316
**H**	0.6967	0.76234	0.59992
**H**	0.88678	0.56509	0.59582
**H**	0.81977	0.34505	0.50418
**H**	0.84247	0.50256	0.44009
**H**	0.39939	0.48492	0.55095
**H**	0.5598	0.53988	0.56871

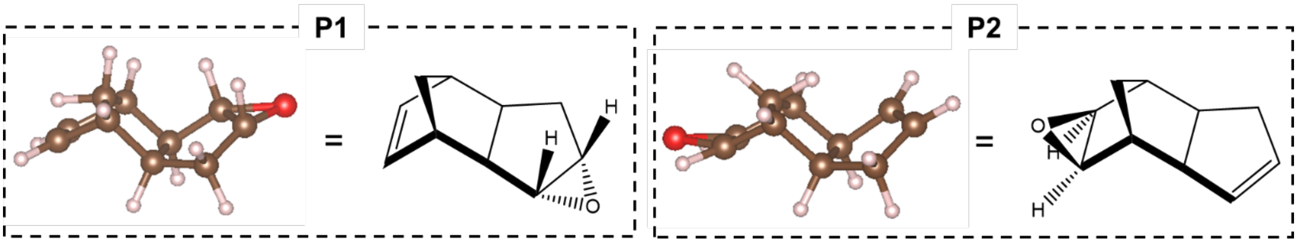

Figure S7. Optimized P1 and P2 diastereomeric products of epoxidation reactions on TiO_2 after DFT calculations. Regardless of the DCPD approaching direction to Ti-O_2*, fully optimized structures in DFT calculations are obtained as the illustrated chemical structure.

References

1 Y. Kim, H. M. Hwang, L. Wang, I. Kim, Y. Yoon and H. Lee, *Sci. Rep.*, , DOI:10.1038/srep25212.

2 R. A. Spurr and H. Myers, *Anal. Chem.*, 1957, **29**, 760–762.

3 G. Kresse and J. Furthmüller, *Comput. Mater. Sci.*, 1996, **6**, 15–50.

4 R. A. Vargas-Hernández, *J. Phys. Chem. A*, 2020, **124**, 4053–4061.

5 J. P. Perdew, K. Burke and M. Ernzerhof, *Phys. Rev. Lett.*, 1996, **77**, 3865–3868.

6 C. J. Bartel, A. W. Weimer, S. Lany, C. B. Musgrave and A. M. Holder, *npj Comput. Mater.*, 2019, **5**, 4.

7 D. Pan and G. Galli, *Nat. Commun.*, 2020, **11**, 421.

8 D. Joubert, *Phys. Rev. B - Condens. Matter Mater. Phys.*, 1999, **59**, 1758–1775.

9 P. E. Blöchl, *Phys. Rev. B*, 1994, **50**, 17953–17979.

10 S. Grimme, J. Antony, S. Ehrlich and H. Krieg, *J. Chem. Phys.*, , DOI:10.1063/1.3382344.

11 H. Zhang, M. Yu and X. Qin, *Nanomaterials*, 2019, **9**, 535.

12 M. Nag, S. Ghosh, R. K. Rana and S. V. Manorama, *J. Phys. Chem. Lett.*, 2010, **1**, 2881–2885.
